# Emphysema severity-associated gut microbiota modulates smoke-induced emphysema: evidence from fecal microbiota transplantation

**DOI:** 10.1186/s12931-026-03743-x

**Published:** 2026-06-04

**Authors:** Na Hyun Kim, Jang Ho Lee, Jaeik Oh, Sunyoung Lee, Eun Sung Jung, Dong Ho Suh, Hye-Ji Kang, Bobae Kim, Hye-Shin Kim, Heeseo Kim, I Na Rae Yun, Eun Hye Kim, Eunsil Kim, Jin-Yong Jeong, Yosep Ji, Sung-Yup Cho, Sei Won Lee

**Affiliations:** 1 Department of Pulmonology and Critical Care Medicine, Asan Medical Center, University of Ulsan College of Medicine, 88 Olympic-ro 43-gil, Songpa-gu, Seoul, 05505 Republic of Korea; 2 Medical Research Center, Genomic Medicine Institute, Seoul National University College of Medicine, Seoul, Republic of Korea; 3 HEM Pharma Inc, Suwon, Republic of Korea; 4 Department of Convergence Medicine, Asan Institute for Life Sciences, Asan Medical Center, University of Ulsan College of Medicine, Seoul, Republic of Korea; 5 Department of Biomedical Sciences, Seoul National University College of Medicine, Seoul, Republic of Korea; 6 Medical Research Center, Genomic Medicine Institute, Seoul National University College of Medicine, Seoul, Republic of Korea; 7 Cancer Research Institute, Seoul National University, Seoul, Republic of Korea

**Keywords:** Chronic obstructive pulmonary disease, Emphysema, Microbiome, Fecal microbiota transplantation, Gut-lung axis

## Abstract

**Background:**

Cigarette smoking is the key risk factor for chronic obstructive pulmonary disease, but even similar levels of smoking can result in different disease severity. We hypothesize that differences in gut microbiota and metabolites contribute to differences in emphysema severity through the gut–lung axis. In this study, we compared the microbiome and metabolome among non-emphysema, non-severe emphysema and severe emphysema groups. Additionally, the impact of fecal microbiota transplantation from non-emphysema, non-severe emphysema and severe emphysema groups on emphysema were investigated.

**Methods:**

A total of 78 participants with a smoking history were included in this study and categorized into three groups: non-emphysema, non-severe emphysema, and severe emphysema. Gut microbiota and metabolites were analyzed, and germ-free mice underwent fecal microbiota transplantation with feces from donors representative of each group prior to smoking exposure.

**Results:**

Significant differences in gut microbiota and metabolites were observed among the groups, with lower acetic acid levels in patients with severe emphysema, and a greater abundance of *Prevotellaceae* and *Megasphaera* in patients without emphysema. Fecal microbiota transplantation from donors with severe emphysema worsened lung pathology in mice subjected to smoking exposure, whereas fecal microbiota transplantation from donors without emphysema attenuated emphysema development.

**Conclusions:**

Gut microbiota and metabolites in participants with a smoking history differ according to the presence of emphysema and its severity, and can affect emphysema development. This suggests a role for gut microbiota in lung disease and provides a foundation for exploring gut microbiota as a potential therapeutic target for chronic obstructive pulmonary disease.

**Supplementary Information:**

The online version contains supplementary material available at 10.1186/s12931-026-03743-x.

## Background

Chronic obstructive pulmonary disease (COPD) is characterized by progressive and irreversible airflow limitation and carries a high disease burden worldwide [1]. In recent decades, bronchodilators have represented the primary pharmacological treatment for COPD [2]. However, bronchodilation therapy cannot cure or prevent COPD, and has still limited treatment benefits, especially in emphysema dominant phenotype [1, 3, 4]. More effective therapeutic strategies are therefore required both before and after COPD development.

Although several etiotypes for COPD have recently been proposed, cigarette smoking remains the key environmental risk factor [1]. Individuals vary in their sensitivity to smoking [5], with this variability partially explained by factors such as exercise, diet, and genetic predisposition [6–8]. Epidemiological studies have revealed that a high-fiber diet can provide protection against smoking or air pollution [6, 9, 10]; however, the exact mechanism underlying this protection remains unclear. Several previous studies on the gut–lung axis have shown that the beneficial effects of fiber can be associated with gut microbiota metabolism [11].

We therefore hypothesized that individual differences in emphysema development and progression following similar smoking exposures are related to gut microbiota and its metabolites. To test this hypothesis, we analyzed the microbiome and metabolome of participants with a smoking history in conjunction with emphysema and lung function. Based on this analysis, feces from representative participants in each group were transplanted into germ-free (GF) mice to investigate the effects of gut microbiota and metabolites on emphysema development.

## Methods

### Study population

Individuals aged over 18 years with a smoking history were considered for inclusion in this study. Those who gave informed consent to participate in the study and to provide a fecal and sputum sample between January 2020 and April 2021 were enrolled in the study.

Participants were initially classified into two groups according to the presence of emphysema on chest computed tomography (CT) at enrollment, as determined by two independent pulmonologists. In cases of disagreement, a consensus was reached after discussion. Participants with emphysema were then further classified into two groups according to the forced expiratory volume in one second (FEV_1_): ≥50% of predicted normal value (moderate obstruction) and < 50% of predicted normal value (severe and very severe obstruction) [1]. Finally, participants were classified into three groups: without emphysema (non-emphysema group), emphysema with moderate airflow limitation (non-severe emphysema group), and emphysema with severe airflow limitation (severe emphysema group). For fecal microbiota transplantation (FMT) into GF mice, two donors from each clinical group were specifically selected to ensure sample integrity, biosafety, and group representativeness. The selection criteria included reliable compliance with stool donation, proximity to the hospital to enable immediate transport and anaerobic processing, absence of microbiological findings unsuitable for introduction into the animal facility, and microbiome profiles located near the centroid of the respective group on beta-diversity ordination. Individuals exposed to antibiotics within the previous two months and those with underlying diseases such as malignancy, congestive heart failure, or diabetes mellitus treated with insulin were excluded from the study. The study was approved by the Institutional Review Boards of the Asan Medical Center (no. 2020 − 0314). All patients were informed of the aim and procedure of the study and provided written informed consent before enrollment. This study was conducted in accordance with the Declaration of Helsinki. The detailed procedures for sample preparation and FMT are provided in the Online Supplementary Information.

### Bacterial metagenomic analysis and metabolome measurement

DNA was extracted from fecal and sputum samples for metagenomic analysis. Bacterial genomic DNA was amplified using 16S_V3_F and 16S_V4_R primers (feces) or 16S_V4_F and 16S_V4_R primers (sputum). Short chain fatty acid (SCFA) levels were also measured in fecal samples. Detailed experimental and analysis methods are provided in the Online Supplementary Information. The sequencing data have been deposited in the NCBI BioProject repository under accession number PRJNA1307239. The metabolomics data have been deposited to the MetaboLights repository with the study identifier MTBLS12899 [12].

### Murine model of emphysema

All animal care and experimental procedures were approved by the Institutional Animal Care and Use Committee of the Asan Medical Center (Permit no. 2022-13-126) and Handong Global University (Permit no. IACUC20221026-14). Male C57BL/6 GF mice (6 weeks old) obtained from CLEA Japan Inc. (Tokyo, Japan) underwent FMT with samples from representative donors in each group. No antibiotic pretreatment was used because recipient mice were maintained GF before colonization. Donor stool was freshly suspended under anaerobic conditions (1 g in 10 mL sterile PBS), filtered through a 70-µm filter, and administered via oral gavage (200 µL per mouse) once weekly for 3 consecutive weeks using donor-matched inocula. Smoking exposure was initiated after the completion of FMT. Age-matched SPF C57BL/6 mice were used as controls or for smoking exposure without FMT. Mice in the smoking exposure groups were exposed to cigarette smoke in a whole-body apparatus five days a week for four weeks, using 12 commercial cigarettes per day (four cigarettes/session, three sessions/day, 8.0 mg tar/cigarette, and 0.70 mg nicotine/cigarette; Camel, R. J. Reynolds Tobacco Company, Winston-Salem, NC, USA). All mice were exposed to smoking using the same apparatus simultaneously starting at 11 weeks of age. During the four-week smoking exposure period, poly I: C (polyinosinic: polycytidylic acid) was intranasally administered at a dose of 50 µL/mouse, twice a week in the third and fourth weeks [13]. The control group inhaled only clean-room air. A total of 4 weeks of experiments were conducted and mice were sacrificed. Emphysema was assessed by calculating the mean linear intercept (MLI) on histopathology images [14]. Further details are provided in the Online Supplementary Information.

### Statistical analysis

Clinical data are presented as mean ± standard deviation for continuous variables and number (percentage) for categorical variables. While the Student’s *t*-test, Kruskal–Wallis test, and one-way analysis of variance, followed by Tukey’s test, for continuous variables, the *χ*^2^ or Fisher’s exact test were used for categorical variables to assess significant differences. *P* < 0.05 was considered to indicate statistical significance. The ASV feature table and taxonomic classification data were processed using phyloseq package in R (Version 4.3.0). The Total count normalization was performed with Total Sum Scaling (TSS). Statistical analyses were performed using nonparametric tests (Wilcoxon rank-sum test) and permutational multivariate ANOVA(PERMANOVA). All analyses were performed using SPSS software, version 24.0 (IBM Corp., Armonk, NY, USA) and R statistical software, version 3.6.3 (R Foundation for Statistical Computing, Vienna, Austria). Detailed analysis method of the experimental results was described in the Online Supplementary Information.

## Results

### Participant characteristics

A total of 78 people with a smoking history were included in the study, 19 in the non-emphysema group, 26 in the non-severe emphysema group, and 33 in the severe emphysema group (Figure S1). Table 1 presents the baseline participant characteristics. There were no differences among the groups in terms of age, sex, smoking amount, or COPD exacerbation history in the previous year. However, patients in severe emphysema group exhibited significantly lower body mass index, pulmonary function parameters, and COPD assessment test scores than the other groups.

**Table 1:** Baseline participant characteristics

Characteristic	Total (*n* = 78)	Non-emphysema (*n* = 19)	Non-severe emphysema (*n* = 26)	Severe emphysema (*n* = 33)	*P*-value
Male	72 (92.3%)	18 (94.7%)	24 (92.3%)	30 (90.9%)	0.883
Age, years	66.1 ± 6.1	64.6 ± 6.1	66.9 ± 6.2	66.3 ± 6.1	0.419
Body mass index, kg/m^2^	22.6 ± 3.4	24.9 ± 2.8	23.9 ± 3.2	20.3 ± 2.5	< 0.001
Current smoking status	67 (85.9%)	12 (63.2%)	23 (88.5%)	32 (97.0%)	0.003
Smoking amount, pack-years	42.3 ± 16.4	43.2 ± 17.2	40.8 ± 10.9	43.0 ± 19.7	0.761
Exacerbation in the previous year	6 (7.7%)	0 (0.0%)	2 (13.3%)	4 (26.7%)	0.112
COPD assessment test score	15.8 ± 9.1	9.7 ± 4.3	14.5 ± 9.8	23.2 ± 6.7	< 0.001
Pulmonary function test					
FEV_1_ measured, L	1.91 ± 0.98	2.78 ± 0.54	2.54 ± 0.59	0.92 ± 0.28	< 0.001
FEV_1_% predicted	61.7 ± 29.4	87.1 ± 13.2	82.4 ± 15.9	30.8 ± 8.2	< 0.001
FVC measured, L	3.60 ± 0.76	3.92 ± 0.57	4.01 ± 0.68	3.08 ± 0.61	< 0.001
FVC % predicted	85.4 ± 14.2	89.3 ± 10.0	93.9 ± 13.2	76.5 ± 12.0	< 0.001
FEV_1_/FVC	51.1 ± 20.3	70.9 ± 7.9	63.5 ± 10.3	29.9 ± 7.0	< 0.001
DL_CO_ measured, mL/min/mmHg	12.2 ± 6.3	18.0 ± 3.6	15.3 ± 4.8	6.2 ± 2.6	< 0.001
DL_CO_ % predicted	57.7 ± 28.0	82.9 ± 14.1	72.2 ± 20.7	31.0 ± 12.8	< 0.001

Data are presented as mean ± standard deviation or number (%). The Kruskal–Wallis test was used to compare continuous variables and the χ^2^ was used to compare categorical variables

Abbreviations: *COPD* chronic obstructive pulmonary disease, *DLco* carbon monoxide diffusion in the lung, *FEV*_*1*_ forced expiratory volume in 1 s, *FVC* forced vital capacity

The levels of six SCFAs were measured in fecal samples (Table 2), revealing that acetic acid levels were significantly decreased in the non-severe (53.03 ± 23.16 µmol/g) and severe (60.37 ± 20.42 µmol/g) emphysema groups compared to the non-emphysema group (71.01 ± 18.10 µmol/g, *P* = 0.006). Propionic acid (18.59 ± 5.72 µmol/g in non-emphysema group vs. 16.76 ± 14.63 µmol/g in non-severe emphysema group vs. 15.55 ± 7.01 µmol/g in severe emphysema group, *P* = 0.059) and butyric acid (13.51 ± 5.33 µmol/g in non-emphysema group vs. 9.87 ± 4.27 µmol/g in non-severe emphysema group vs. 10.66 ± 5.59 µmol/g in severe emphysema group, *P* = 0.056) exhibited similar trends.

**Table 2:** Comparison of fecal short-chain fatty acid levels among participants with varying severity of emphysema

Short chain fatty acid	Non-emphysema (*n* = 19)	Non-severe emphysema (*n* = 26)	Severe emphysema (*n* = 33)	*P*-value
Acetic acid (µmol/g)	71.01 ± 18.10	53.03 ± 23.16	60.37 ± 20.42	0.006
Propionic acid (µmol/g)	18.59 ± 5.72	16.76 ± 14.63	15.55 ± 7.01	0.059
Isobutyric acid (µmol/g)	1.46 ± 0.98	1.58 ± 0.98	1.61 ± 1.06	0.871
Butyric acid (µmol/g)	13.51 ± 5.33	9.87 ± 4.27	10.66 ± 5.59	0.056
Isovaleric acid (µmol/g)	2.05 ± 1.83	2.39 ± 1.87	2.49 ± 1.84	0.558
Valeric acid (µmol/g)	1.79 ± 1.19	1.84 ± 1.36	1.88 ± 1.63	0.971

Data are presented as mean ± standard deviation. The Kruskal–Wallis test was used for analysis. Acetic acid levels were significantly decreased in the non-severe and severe emphysema groups compared to the non-emphysema group. Propionic acid and butyric acid exhibited similar trends

### Microbiome analysis

**Fig. 1:**
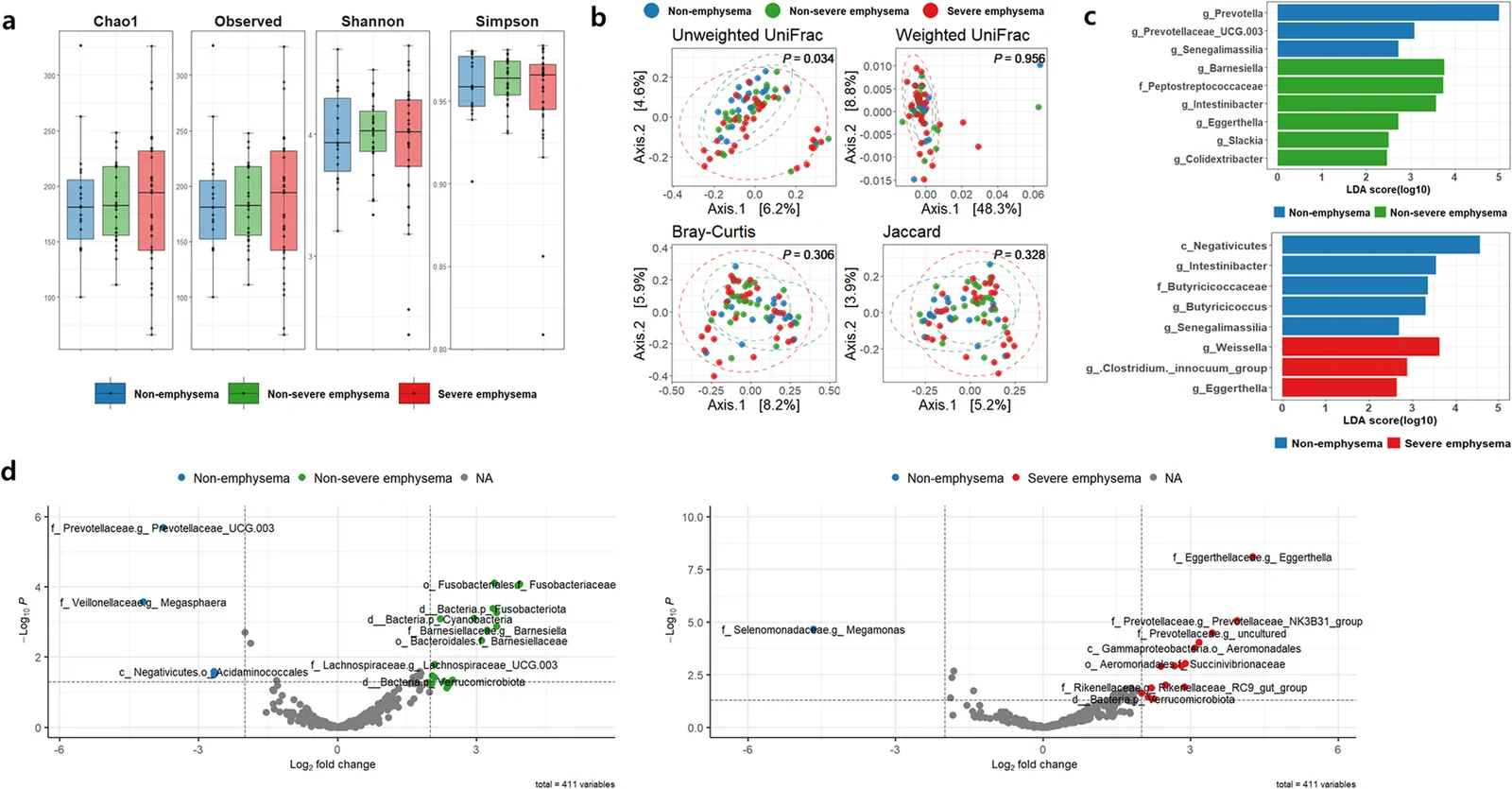
Gut microbiome characteristics of the three groups. **a** Boxplots of alpha-diversity indices (Chao 1, Observed, Shannon, and Simpson Indices). No significance difference was noted between groups (Wilcoxon rank sum test). **b** Beta-diversity indices (PERMANOVA, permutation = 999). Significant differences of beta-diversity were reported in unweighted UniFrac. **c** LDA effect size. Bacteria of *P* < 0.05 (Wilcoxon rank sum test) and LDA score > 2 was presented. **d** Volcano plot showing differential bacterial abundance (DESeq2 analysis). Bacteria of adjusted *P* < 0.05 and Log_2_ fold change > 2 was presented. *Prevotellaceae*, *Megasphaera*, and *Acidaminococcales* were more prevalent in the non-emphysema group than in the non-severe emphysema group. *Megamonas* was more common in the non-emphysema group than in the severe emphysema group. Abbreviation: *LDA*, linear discriminant analysis

Figure 1 presents the gut microbiome characteristics of the three groups. No significant differences were observed in the alpha-diversity indices among the three groups (Fig. 1a); however, among the four prespecified beta-diversity indices, unweighted UniFrac was significant (*P* = 0.034, Fig. 1b), suggesting a phylogenetically structured presence/absence turnover rather than a broad abundance shift. Figure 1c shows the bacterial groups that were differentially abundant between the groups, as determined by a linear discriminant analysis score > 2.0. Volcano plots (Fig. 1d) revealed that *Prevotellaceae*, *Megasphaera*, and *Acidaminococcales* were more prevalent in the non-emphysema group than in the non-severe emphysema group. A separate analysis of the respiratory microbiome using sputum was performed; the findings, detailed in the supplement, provided an interesting contrast to those of the gut. While the gut microbiome did not show a clear trend in alpha-diversity, the sputum microbiome did: alpha-diversity tended to decrease with increased emphysema severity (Figure S2). Furthermore, this severity-dependent shift was characterized by an increased abundance of pathobionts, such as *Enterobacteriaceae* and *Moraxellaceae*, in the severe emphysema group (Figures S2-4).

**Fig. 2:**
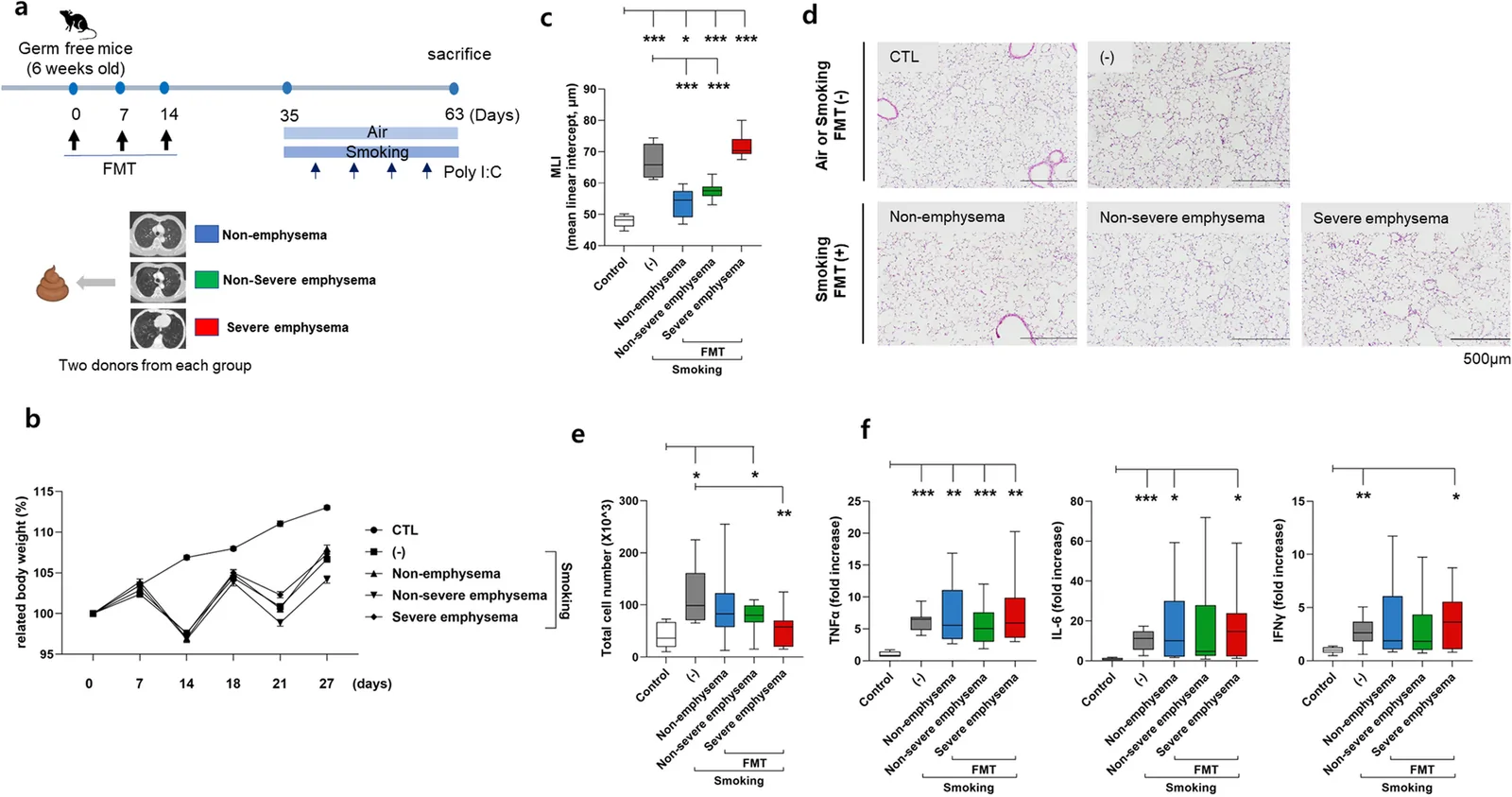
Emphysema development in germ-free mice after smoking exposure following FMT. **a** Experimental design of germ free mice. **b** Body weight changes since smoking exposure. **c** Emphysema development assessed by calculating the MLI of lung tissue. **d** Hematoxylin and eosin staining of left lung histology. Representative images are shown. Scale bar = 500 μm (magnification: ×100). Compared to the control group, the smoking exposure without FMT group showed increased MLI. Increased MLI was reversed in the non-emphysema and non-severe emphysema groups, not in severe emphysema group **e** Total number of cells in the bronchoalveolar lavage fluid. Inflammatory cell counts in bronchoalveolar lavage fluid were lower in severe emphysema group mice than in smoking exposure without FMT group mice (**f**) Relative mRNA levels of TNF-α, IL-6, and IFN-γ in lung tissues. Two-sample *t* test was used. All box and whisker plots illustrates the median, the interquartile range and the largest and smallest observed values. *P < 0.05, **P < 0.01, and ***P < 0.001. (-) indicates the smoking exposure without FMT group. Abbreviations: *FMT*, fecal microbiota transplantation; *IFN*, interferon; *IL*, interleukin; *MLI*, mean linear intercept; *TNF*, tumor necrosis factor

**Fig. 3:**
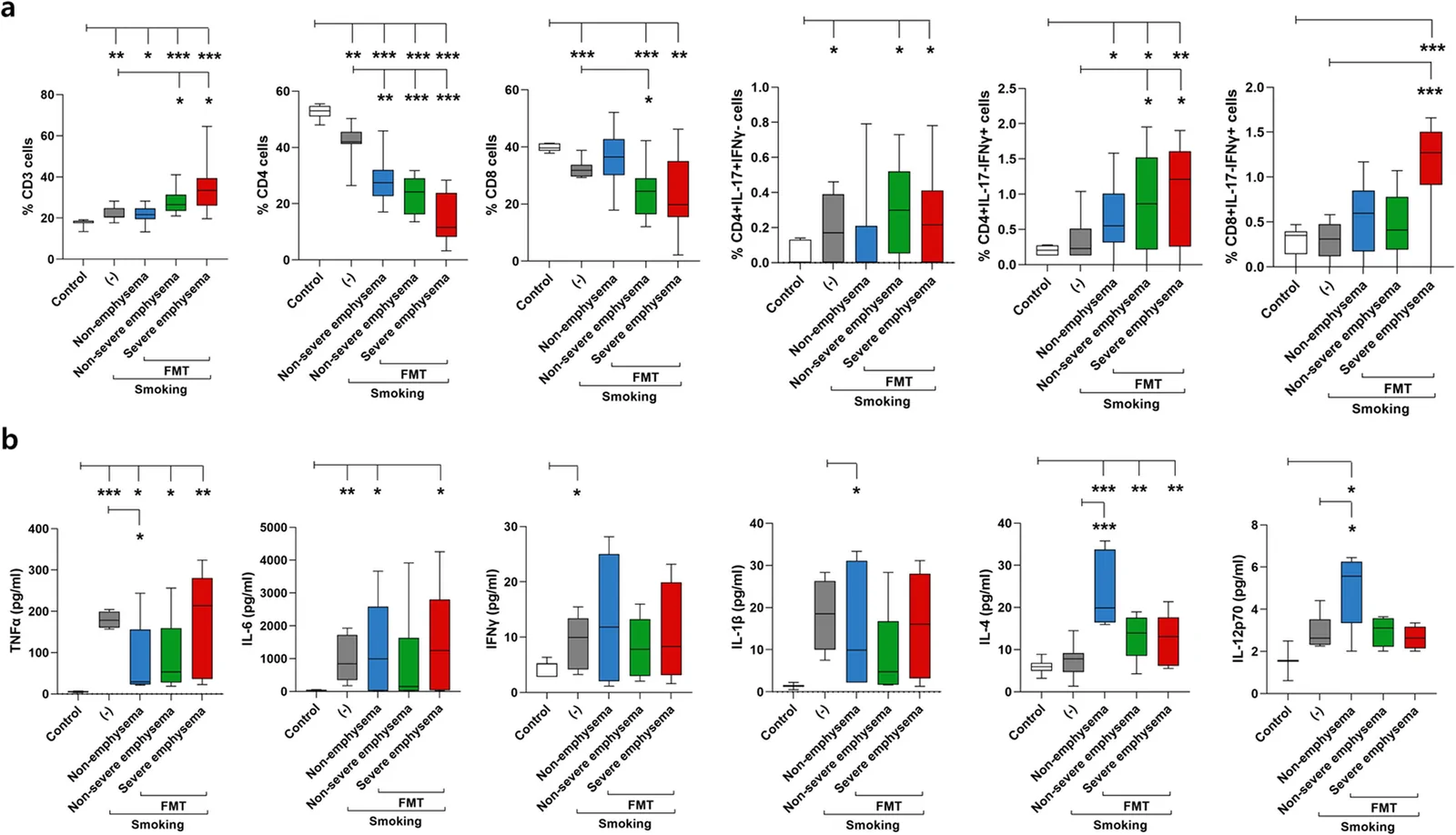
Changes of inflammatory cell proportions and cytokine production in germ-free mice after smoking exposure following FMT. **a** Percentages of CD3^+^, CD4^+^, CD8^+^, CD4^+^IL-17^+^, CD4^+^IFN-γ^+^, and CD8^+^IFN-γ^+^ T cells in the lung tissue of mice. The proportion of CD3 + cells was increased in mice exposed to smoking compared with control group mice and highest in the severe emphysema group. The proportions of CD4 + IL-17 + IFN-γ-, CD4 + IL-17-IFN-γ+, and CD8 + IL-17-IFN-γ + cells were significantly higher in the lungs of severe emphysema group mice than in control group mice, and the latter two T cell types were also significantly higher in severe emphysema group mice than in mice exposed to smoking without FMT (**b**) Levels of TNF-α, IL-6, IFN-γ, IL-1β, IL-4, and IL-12p70 in BALF of mice. TNF-α and IL-1β levels in BALF increased in the smoking exposure without FMT group compared to the control group, but were reversed in the non-emphysema group. Two-sample *t* test was used. Data are presented as mean values with SD. All box and whisker plots illustrates the median, the interquartile range and the largest and smallest observed values. *P < 0.05, **P < 0.01, and ***P < 0.001. (-) indicates the smoking exposure without FMT group. Abbreviations: *BALF*, bronchoalveolar lavage fluid; *FMT*, fecal microbiota transplantation; *IFN*, interferon; *IL*, interleukin; *TNF*, tumor necrosis factor

**Fig. 4:**
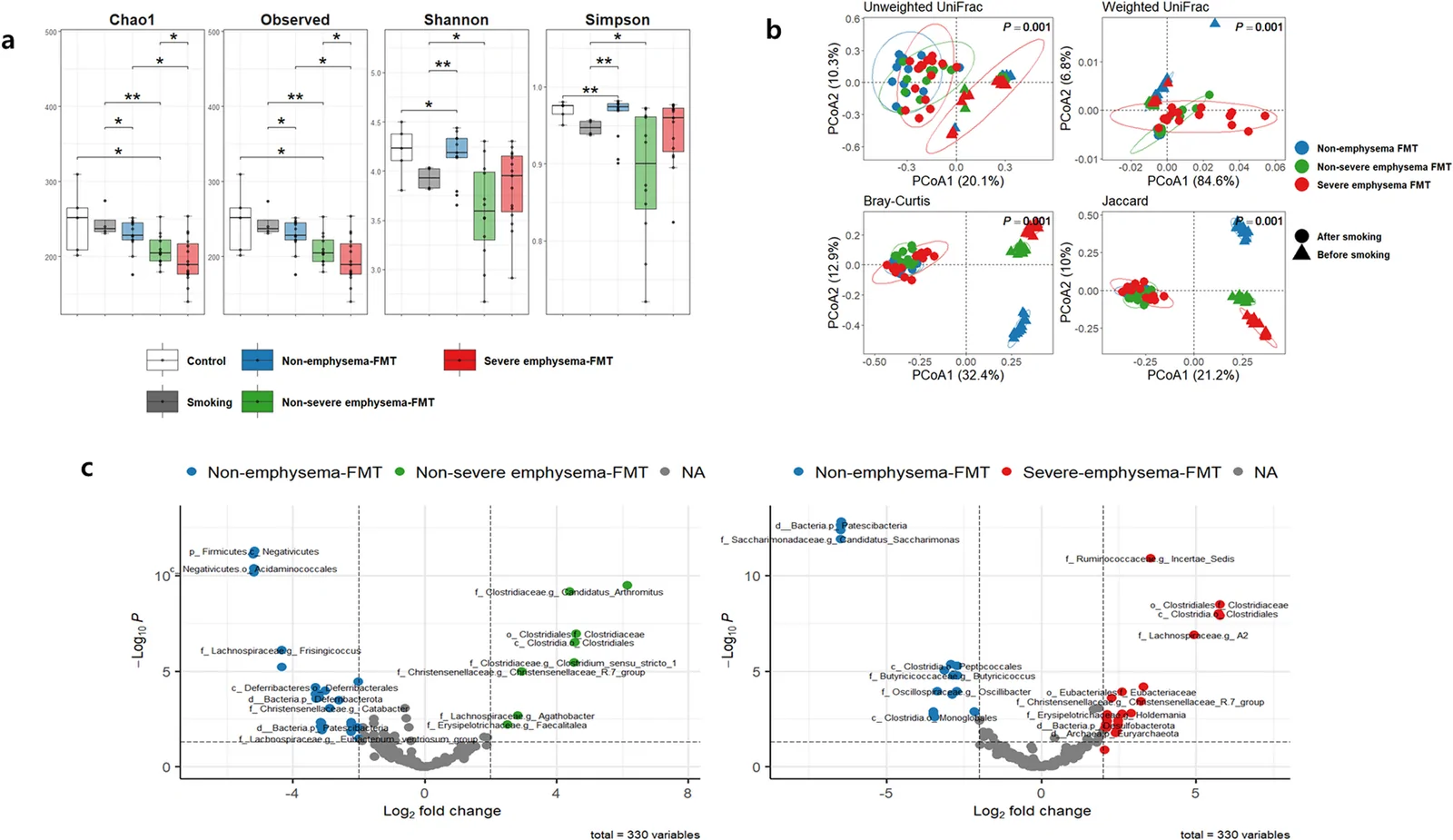
Gut microbiome of germ-free mice after smoking exposure following FMT. **a** Box plots of alpha-diversity indices (Chao 1, Observed, Shannon, and Simpson Indices). Wilcoxon rank sum test was used. *P < 0.05, **P < 0.01. Alpha-diversity indices were significantly different in non-emphysema and non-severe emphysema group mice compared with severe emphysema group mice. **b** Beta-diversity indices (PCoA of unweighted UniFrac, weighted UniFrac, Bray–Curtis, and Jaccard Indices). *P*-value was calculated by PERMANOVA (permutation = 999). Significant differences of beta-diversity were reported in all beta-diversity indices. **c** Volcano plot showing differential bacterial abundance (DESeq2 analysis). Bacteria of adjusted *P* < 0.05 and Log_2_ fold change > 2 was presented. Bacterial abundance significantly different between groups. The *Acidaminococcales* order, the dominant bacterial group in human fecal samples from the non-emphysema group, was also dominant in non-emphysema group mice. **d** Box plots of the short chain fatty acid levels in the five groups. *P < 0.05 and **P < 0.01 by ANOVA, post hoc: Tukey. Acetic acid levels in the fecal samples were lower in the severe emphysema group than in the non-emphysema and control groups. Abbreviations: *FMT*, fecal microbiota transplantation; *PCoA*, Principal Coordinates Analysis

### Emphysema development after fecal microbiota transplantation

Animal experiments were conducted to explore the impact of the gut microbiome on emphysema development. GF mice underwent FMT using fecal samples from two representative donors from each group (Figure S5). Table 3 presents the clinical characteristics of FMT donors, who were aged 61–72 years and had a smoking history of 25–60 pack-years; all were male. Pulmonary function parameters were normal in donors from the non-emphysema group, but the FEV_1_ and diffusing capacity of the two donors from the severe emphysema group were 22% and 31%, and 14% and 36% of predicted normal values, respectively, despite the greater smoking amounts of the non- emphysema group donors.

**Table 3:** Characteristics of fecal microbiota transplantation donors

Characteristic	Non-emphysema group		Non-severe emphysema group		Severe emphysema group	
	Donor 1	Donor 2	Donor 3	Donor 4	Donor 5	Donor 6
CT imaging						
Sex/Age, years	Male/72	Male/62	Male/65	Male/70	Male/61	Male/68
Body mass index, kg/m^2^	28.7	27.1	22.5	28.7	18.5	17.8
Smoking status	Former	Former	Current	Former	Former	Former
Smoking amount, pack-years	50	60	25	53	40	40
Pulmonary function test						
FEV_1_ measured, L	2.67	3.08	2.99	3.04	0.68	0.82
FEV_1_% predicted	89	92	97	85	22	31
FVC measured, L	3.58	3.86	4.01	3.77	3.06	3.21
FVC % predicted	80	85	96	72	71	91
FEV_1_/FVC	75	80	75	81	22	26
DL_CO_, mL/min/mmHg	19.8	20.4	15.5	22.4	2.9	6.3
DL_CO_ % predicted	98	90	75	91	14	36

Abbreviations: *CT* computed tomography, *DLco* carbon monoxide diffusion in the lung, *FEV*_1_ forced expiratory volume in 1 s, *FVC* forced vital capacity

Figure 2a illustrates the experimental design. Mice exposed to smoking experienced a reduction in body weight compared with the control group. Body weights were measured from the start of smoking exposure after FMT (Fig. 2b). Compared to mice in the severe emphysema group, mice in the non-emphysema and non-severe emphysema groups exhibited significantly decreased MLI (Fig. 2c and d). In smoking-exposed groups, inflammatory cell counts in bronchoalveolar lavage fluid (BALF) were lower in severe emphysema group mice than in smoking exposure without FMT group mice (Fig. 2e). In addition, mRNA expression levels of the proinflammatory mediators, including tumor necrosis factor (TNF)-α, interleukin (IL)-6, and interferon gamma (IFN)-γ, were elevated in mice exposed to smoking compared with control group mice (Fig. 2f). The proportion of CD3^+^ cells increased in mice exposed to smoking compared with control group mice and highest in the severe emphysema group. The proportions of CD4^+^IL-17^+^IFN-γ^−^, CD4^+^IL-17^−^IFN-γ^+^, and CD8^+^IL-17^−^IFN-γ^+^ cells were significantly higher in the lungs of severe emphysema group mice than in control group mice, and the latter two T cell types were also significantly higher in severe emphysema group mice than in mice exposed to smoking without FMT (Fig. 3a). TNF-α and IL-1β levels were decreased in BALF from non-emphysema group mice compared with smoking exposure without FMT group mice (Fig. 3b).

### Gut microbiome and metabolites of mice following FMT

Feces were collected from the colon of GF mice following FMT and smoking exposure for microbiome and metabolite analysis. Alpha-diversity indices were significantly different in non-emphysema and non-severe emphysema group mice compared with severe emphysema group mice (Fig. 4a). Figure 4b illustrates the change in beta-diversity indices before and after smoking. Bacterial abundance differed significantly between the non-emphysema group and the non-severe and severe emphysema groups (Fig. 4c). The *Acidaminococcales* order, which was the dominant bacterial group in human fecal samples from the non-emphysema group, was also dominant in non-emphysema group mice. The gut microbiome as measured in fecal samples included abundant bacteria and is described in more detail in Figure S6 and S7.

**Fig. 5:**
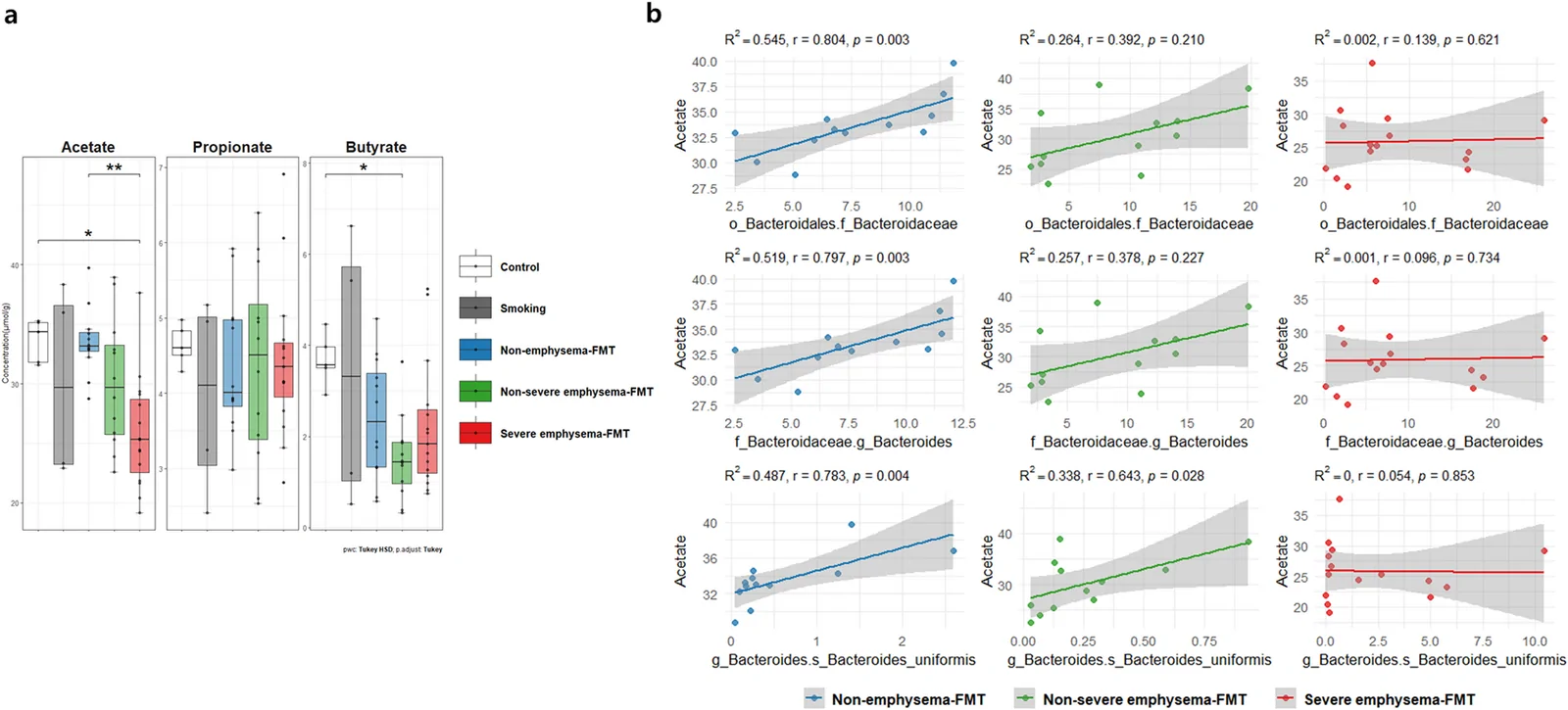
Metabolomic and transcriptomic profiles in germ-free mice after FMT and smoking exposure. **a** Fecal levels of short-chain fatty acids (SCFAs)—acetate, propionate, and butyrate—measured at sacrifice in germ-free mice exposed to smoking after FMT from donors with varying emphysema severity. Acetic acid levels were significantly higher in the non-emphysema-FMT group compared with the severe emphysema-FMT group and the smoking-only group. (b) Correlation analysis between fecal acetate levels and the abundance of *Bacteroides uniformis*, and relevant family and genera, identified based on microbiome–metabolome integration. Abbreviations: *FMT*, fecal microbiota transplantation

Measurement of fecal SCFA levels at sacrifice revealed that acetic acid levels were significantly lower in the severe emphysema group than in the non-emphysema and control groups, consistent with the results of the human analysis (Fig. 5a). To investigate which bacterial taxa were associated with elevated acetate levels observed in the non-emphysema group, we conducted an integrated microbiome–metabolome correlation analysis. As shown in Figure S8, the relative abundance of the *Bacteroides* genus, and particularly *Bacteroides uniformis* at the species level, was positively correlated with fecal acetate concentrations. To further validate this finding, we performed a linear regression analysis between acetate levels and the abundance of *Bacteroides uniformis*, which confirmed a strong positive association (Fig. 5b). These results suggest that *Bacteroides uniformis* may contribute to acetate production and play a role in the protective effects against emphysema observed in the non-emphysema group. Although SCFA supplementation was not directly tested in the present donor-specific FMT experiment, several preclinical emphysema models support the causal potential of SCFAs in attenuating smoke-induced lung injury [15–17].

### RNA sequencing of mice following FMT

**Fig. 6:**
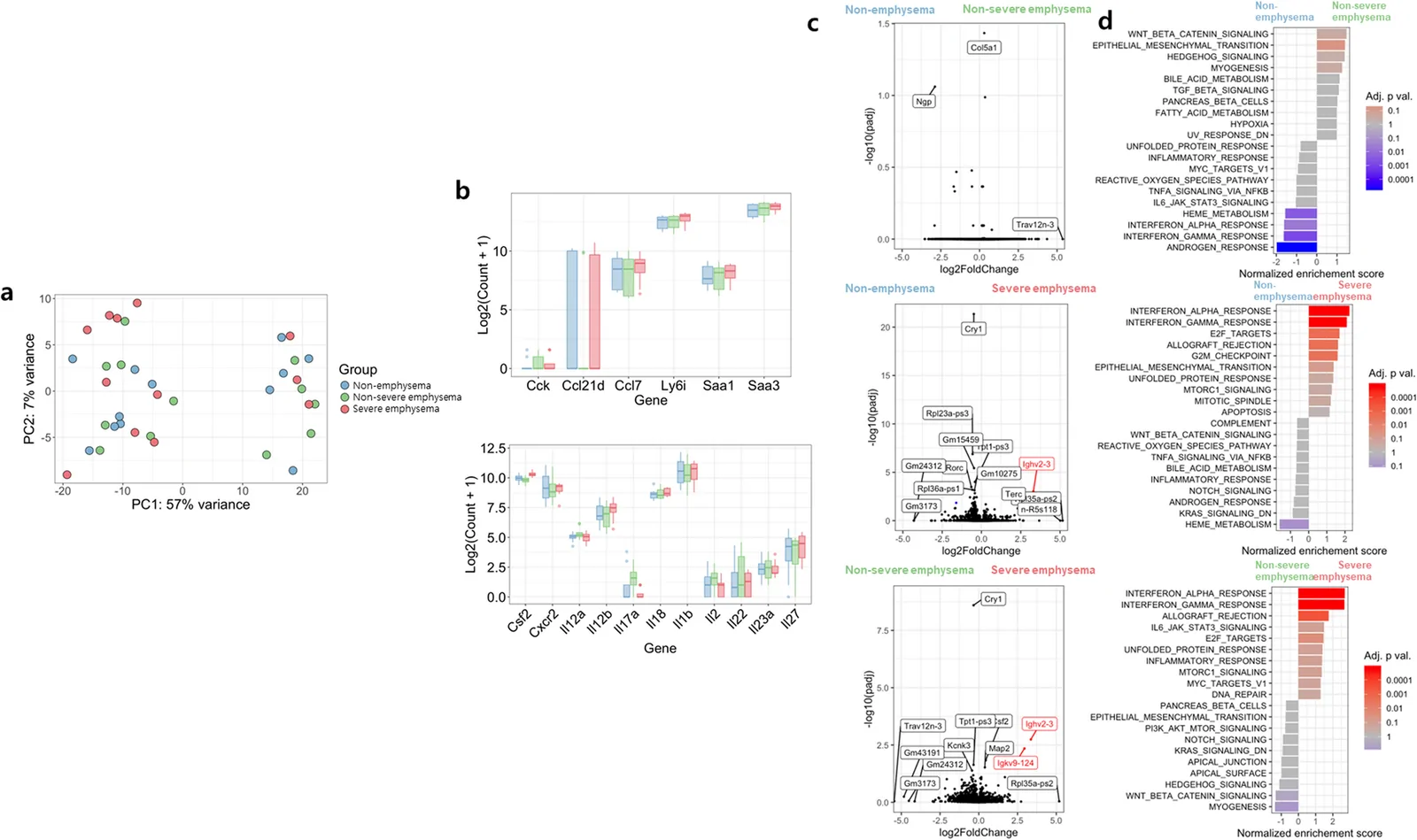
Bulk RNA sequencing of mice lung tissue after smoking exposure following FMT. **a** Principal component analysis (Deseq2). **b** Cytokine expression normalized by log_2_Count. **c** Volcano plots of differentially expressed genes. Significantly differentially expressed genes are presented (adjusted *P* < 0.05 and Log2FC > 0.5). The expression of *Gm37173*, *Tpt1-ps3*, *Rpl35a-ps2*, and *Cry1* was decreased in the severe emphysema group compared to the non-emphysema and non-severe emphysema groups **(d)** Bar plot of gene set enrichment analysis derived from hallmark gene set. Upper and lower 20 sets were presented. Logarithmic adjusted *P*-value is indicated by color. Immune-related gene sets were upregulated in the order of severe emphysema, non-emphysema, and non-severe emphysema groups. Abbreviations: *FMT*, fecal microbiota transplantation

To examine the specific mechanism underlying emphysema development following FMT, bulk RNA sequencing was performed on five mice per group. No clear division of the groups was observed according to principal component analysis or transcriptome level of cytokines (Fig. 6a and b). The expression of four genes, *Gm37173*, *Tpt1-ps3*, *Rpl35a-ps2*, and *Cry1*, was decreased in the severe emphysema group compared to the non-emphysema and non-severe emphysema groups **(**Fig. 6c**)**. According to gene set enrichment analysis, immune-related gene sets were significantly upregulated in the order of severe emphysema, non-emphysema, and non-severe emphysema groups, and IFN-α and IFN-γ responses were predominant (Fig. 6d).

## Discussion

In this study, we hypothesized that variability in emphysema development and progression after smoking exposure may be related to differences in gut microbiota and metabolites. The results of the study revealed significant differences in gut microbiome composition and SCFA levels among the groups, with lower levels of acetic acid observed in those with emphysema. Moreover, gut microbiome analysis showed distinct bacterial profiles according to the presence and severity of emphysema. The FMT experiments further demonstrated that gut microbiota from individuals with severe emphysema could exacerbate lung pathology in mice, highlighting the potential role of the gut–lung axis in COPD. These findings suggest that gut microbiota and their metabolites may influence the development and progression of emphysema, indicating potential novel therapeutic targets for COPD.

This study primarily focused on the relationship between emphysema and gut microbiota rather than respiratory microbiota, based on the premise that gut microbiota may provide a link between diet and COPD [10, 18], as diet directly influences gut microbiota composition. Analysis of gut microbiota and metabolites revealed that each group was characterized by specific gut bacteria and metabolites. Notably, between-group differences in beta-diversity were observed only in the unweighted UniFrac metric. In contrast, the results for Bray–Curtis, weighted UniFrac, and Jaccard metrics were not significant, suggesting that the observed differences reflect phylogenetically structured turnover in the presence or absence of lineages rather than large shifts in dominant taxa. For example, acetic acid, a product of dietary fiber fermentation by gut microbiota, was found at significantly higher concentrations in participants without emphysema than in those with emphysema. Propionate and butyrate, two other SCFAs, also showed similar trends. Previous studies have identified these SCFAs as having protective roles against emphysema development, particularly in the context of smoking exposure [11, 16, 19–21]. These findings are consistent with the study by Trompette et al., which demonstrated that SCFAs play a critical role in modulating airway inflammation [22]. The specific gut bacteria involved in protection against development of emphysema remain unclear, but *Prevotellaceae*, *Megasphaera*, and *Acidaminococcales* were more abundant in participants without emphysema than in those with emphysema. Interestingly, all of these produce SCFAs [23–25]. This suggests that the gut microbiome, shaped by long-term environmental and dietary factors, may differentially influence metabolite formation and, consequently, respiratory disease development. In the respiratory microbiome, *Enterobacteriaceae* and *Moraxellaceae*, which have previously been shown to be increased in COPD exacerbation, were also dominant in severe emphysema [26]. In contrast to the gut-focused results, this finding highlights the complex interplay of the gut–lung axis. For instance, while the gut microbiome did not show a clear trend in alpha-diversity, the sputum microbiome did, with diversity tending to decrease with emphysema severity. This divergence suggests that simple bacterial translocation is insufficient to explain the phenomenon. Instead, a more nuanced view that accounts for bidirectional communication is warranted. A key indirect mechanism, supported by seminal research, is that gut dysbiosis—such as the depletion of SCFA-producing bacteria noted in our study—leads to a compromised systemic immune environment [24]. This systemic immune suppression could, in turn, make distant organs, such as the lungs, more vulnerable to the proliferation of the pathobionts observed. While other pathways, such as microaspiration of altered oral flora, may also contribute, our data support the hypothesis that a dysfunctional gut microbiome contributes to a permissive environment for pathogenic shifts in the lung.

GF mice that received FMT from non-emphysema and non-severe emphysema donors showed attenuated emphysematous changes, as indicated by a reduction in MLI. This finding suggests that factors present in fecal material influence emphysema development. The pivotal link between gut microbiota and lung immune responses has been well-documented, demonstrating that appropriate microbial colonization can attenuate inflammatory damage in the lungs [27]. Although GF mice exhibit altered immune maturation, they provide a controlled gnotobiotic platform to isolate the effect of donor microbiota composition without the confounding influence of a pre-existing resident microbiota. Importantly, our previous study in conventional C57BL/6 mice showed that gut microbiota modulation by FMT and a high-fiber diet attenuated cigarette smoke/poly I: C-induced emphysema. This was accompanied by lower MLI, reduced BALF and serum IL-6 and IFN-γ, and decreased lung inflammatory mediators and apoptosis [15, 28]. Thus, the present donor-specific GF experiment should be interpreted as a complementary model demonstrating donor-dependent effects on the microbiota. At the same time, prior conventional mouse data support the broader biological relevance of gut microbiota modulation in smoke-induced emphysema.

Decreased TNF-α and IL-1β levels in BALF, and altered CD4^+^ and CD8^+^ lymphocyte proportions in lung tissue, were observed alongside the improvement in emphysema, consistent with the results of previous studies that have demonstrated their increase in emphysema and their relationship to emphysema development [29–32]. The exact mechanism underlying this improvement is complex but transfer of beneficial microbiota and metabolites may play a role. Interestingly, *Acidaminococcales* was detected in both the feces of patients without emphysema and mice that received fecal transplants from these patients; acetic acid levels were also high in these mice, and *B. uniformis* was found to have a significant positive correlation with fecal acetate levels in this mice group. This species is a well-characterized commensal bacterium residing in the human gut, known for its metabolic capacity to ferment complex carbohydrates and generate acetate and other SCFAs as end products [33]. Given the complexity of microbial interactions within the gut ecosystem, it is also possible that *B. uniformis* exerts indirect effects on other microbial populations, either promoting or suppressing the abundance of co-resident bacteria through competition for substrates or metabolic cross-feeding [34]. Taken together, even though gut microbiota diversity tends to decrease during smoking exposure, certain characteristics of the gut microbiota may be preserved, influencing susceptibility to emphysema.

Gut microbiota modulation, including FMT, is already utilized in clinical practice [35], and numerous trials have assessed the applicability of this strategy in intestinal diseases, infectious diseases, cancer, and other conditions [36]. Research on its suitability for use in respiratory diseases is still in its early stages and more work is required. The main mechanisms responsible for the effects of microbiome-based therapies involve immune modulation and the restoration of normal flora. In COPD, where airflow limitation and alveolar destruction are the primary pathologies [37], the effects of microbiome modulation may be limited, with a greater impact on the inflammation-related aspects of the disease such as acute exacerbations. SCFAs produced by gut microbiota mediate between the gut microbiome and other organs including the lung [38–40], confirmed in the present study by the variation of acetic acid levels with disease severity in both humans and mice. However, the therapeutic effects of FMT are not limited to SCFA levels, which are affected by several factors including diet, gut microbiome, colonic environment, and gut motility [41]. Another plausible mechanism is that FMT could reinforce the impaired gut integrity in mouse exposed to smoking. Barrier dysfunction is well-documented to be associated with persistent inflammation and various diseases [43]. Gut barrier recovery can mitigate the systemic translocation of pro-inflammatory mediators, thereby reducing distant organ damage, including in the lungs. A previous study demonstrated that exposure to smoking resulted in significant changes to the colonic mucosa with increased bacterial invasion and that interrupting this invasion reduced pulmonary inflammation [13]. Modulating the respiratory microbiome poses challenges, as its symbiosis with the host may be even more delicate than that of the gut microbiome.

This study has certain limitations. First, emphysema severity was classified based on the FEV_1_, not the emphysema index, which could not be utilized because of different CT protocols among the patients. However, FEV_1_ is universally utilized to evaluate the severity of COPD [1], and has shown some correlation with the extent of emphysema [42]. Second, we could not identify the specific bacterial species or mediators from the donor samples responsible for attenuating emphysema. While certain bacterial groups, rather than individual species, may influence emphysema development, further research is necessary to clarify this for future clinical applications. Third, the donor-specific FMT experiment was performed in GF recipients, which have altered immune development. We chose this model to enable controlled assessment of donor microbiota effects without the confounding influence of an established resident microbiota. However, validation in conventional recipients using the present donor set would further strengthen the translational relevance of these findings.

## Conclusions

In conclusion, the differences in gut microbiome and metabolites observed in this study underscore the potential role of the gut–lung axis in the development and progression of emphysema. The findings from FMT experiments suggest a causal role of the gut microbiome in modulating lung pathology in emphysema. These results open new avenues for research into microbiome modulation therapies as a novel approach to COPD treatment.

## Supplementary Information

Supplementary Material 1.

## Data Availability

The sequencing data supporting the conclusions of this study have been deposited in the NCBI BioProject repository under accession number PRJNA1307239. Reviewer access is available via the following link: https://dataview.ncbi.nlm.nih.gov/object/PRJNA1307239?reviewer=t7hvdk9gbijf72cjmc4tmdhkns. The public release date is set for 31 October 2026.The metabolomics data has been deposited to the MetaboLights repository with the study identifier MTBLS12899. Reviewer access is available via https://www.ebi.ac.uk/metabolights/reviewerd7f25502-35e0-4fba-9ba1-cced94e5de77, and the public release date is set for 13 August 2026.

## Abbreviations

BALF: Bronchoalveolar lavage fluid
COPD: Chronic obstructive pulmonary disease
CT: Computed tomography
FEV_1_: Forced expiratory volume in one second
FMT: Fecal microbiota transplantation
GF: Germ-free
IFN: Interferon
IL: Interleukin
MLI: Mean linear intercept
SCFA: Short chain fatty acid
TNF-α: Tumor necrosis factor-α
TSS: Total Sum Scaling
