# Supplementary material for: Emphysema severity-associated gut microbiota modulates smoke-induced emphysema: evidence from fecal microbiota transplantation
Source: Respir Res. 2026 Jun 4;27:316. doi: 10.1186/s12931-026-03743-x (PMC13491976; doi:10.1186/s12931-026-03743-x)
Supplement: Supplementary file 1 [file 12931_2026_3743_MOESM1_ESM.docx]

**Online Supplement**

**Supplementary Methods**

***Human fecal and sputum sample preparation***

Fecal and sputum samples for analysis were collected, snap frozen in liquid nitrogen, and stored at -80°C. For fecal collection, patients were educated in advance to immediately transfer the sample into a sterile collection container after defecation, place it in an insulated pack containing coolant, and bring it to the hospital as soon as possible. All stool samples were processed within four hours of collection. Upon arrival, the samples were aliquoted under anaerobic conditions by a laboratory technician, sealed, and stored in a deep freezer at -80°C until further use. To perform the fecal microbiota transplantation (FMT), six representative participants were selected—two from each group (non-emphysema, non-severe emphysema, and severe emphysema)—based on random selection among those who demonstrated good compliance with stool collection, lived in proximity to the hospital, and showed adequate understanding of the sampling instructions.. The stool samples were aliquoted under anaerobic conditions and stored at -80 ℃ until FMT.

***Measurement of short chain fatty acids***

All short-chain fatty acids (SCFAs) were extracted with 0.2 g of fecal sample in 1 mL of dH_2_O. Homogenized samples were centrifuged at 13,000 rpm for 10 min at 4 ℃ and the supernatant of the centrifuged samples (150 μL) was transferred to 10 mL of screw cap vial with 150 μL of GC buffer solution, which contains (NH_4_)_2_SO_4_, NaH_2_PO_4_, and 2-ethylbutric acid were added as internal standard 1.

Headspace autosampler-gas chromatography-flame ionization detector (HSS-GC-FID) analysis was performed by Agilent 7890B GC system equipped with a 7697A headspace sampler and FID (Agilent Technologies, USA). An HP-innowax capillary column (30 m x 0.32 mm i.d. x 0.50 μL film thickness; Agilent) was used with constant flow of nitrogen as the carrier gas. The operating conditions were as follows; oven temperature, 85 °C; loop temperature 90 °C; transfer lines, 100 °C; FID temperature 250 °C; column temperature was initially at 60 °C, raised to 140 °C at 30 °C/min, then raised to 170 °C at 30 °C, and finally to 180 °C at 40 °C and held for 0.75 min. Data acquisition and operation processing were conducted using ChemStation software (Agilent Technologies). SCFAs were identified and quantified using standard compounds.

***DNA extraction and metagenomic analysis***

DNA was extracted from the fecal samples using Mag-Bind® Universal Pathogen Kit (Omega Bio-tek, Norcross, GA, US). The fecal samples were suspended in 275 μL of SLX-Mlus Buffer, followed by bead beating in a mixermill MM400 (Retsch, Haan, Nordrhein-Westfalen, Germany) with further isolating, cleaning, and eluting procedures followed the manufacturer’s protocols.

The extracted fecal microbial DNA was amplified with 16S Amplicon PCR Forward Primer (5’-TCGTCGGCAGCGTCAGATGTGTATAAGAGACAGCCTACGGGNGGCWGCAG-3’) and 16S Amplicon PCR Reverse Primer (5’-GTCTCGTGGGCTCGGAGATGTGTATAAGAGACAGGACTACHVGGGTATCTAATCC-3’). These fecal samples amplicon primers, 2× KAPA HiFi HotStart ReadyMix (Roche, Basel, Basel-Stadt, Switzerland) and DNA were generated by PCR under conditions of 3 min at 95 °C, followed by 25 cycles at 95 °C for 30 sec, annealing at 55 °C for 30 sec, extension at 72 °C for 30 sec and a final extension at 72 °C for 5 min. Subsequently sample DNAs were cleaned with HiAccuBead (AccuGene, Incheon, Republic of Korea) and a magnetic stand. The Index PCR was performed by a using the IDT indexing primer (Integrated DNA technologies, Coralville, Iowa, US) for Illumina Miseq System, 2× KAPA HiFi HotStart ReadyMix, and PCR grade water. PCR was carried out in a 95 °C for 3 min. 8 cycles of 95 °C for 30 sec, 55 °C for 30 sec, 72 °C for 30 sec, then 72 °C for 5 min and held at 4 °C for PCR reaction. After clean-up step, the concentration of libraries was verified using the Qubit 4.0 (ThermoFisher Scientific, Waltham, Massachusetts, US) with 1× dsDNA HS assay solution (ThermoFisher Scientific) and sequenced using Illumina Miseq system. Reads were sorted using the unique barcodes for each PCR product. The barcode, linker and primer sequences were then removed from the original sequencing reads. The sequencing results was analyzed using Qiime2 bioinformatics pipeline and taxonomic assignment was performed with Silva 138 database.

DNA was extracted using the FastDNA® SPIN Kit for Soil (MP BIO, Cat. No: 6560-200) from sputum sample, in accordance with the manufacturer’s instruction. Sputum DNA was amplified using the 16S_V4_F(5’- TCGTCGGCAGCGTCAGATGTGTATAAGAGACAGGTGCCAGCMGCCGCGGTAA-3’) and 16S_V4_R(5’- GTCTCGTGGGCTCGGAGATGTGTATAAGAGACAGGGACTACHVGGGTWTCTAAT-3’) primers specific for the V4 hypervariable region of the 16S rDNA gene. The amplifications were carried out under the following conditions: initial denaturation at 95 °C for 3min, followed by 25 cycles of denaturation at 95 °C for 30 sec, primer annealing at 55 °C for 30 sec, and extension at 72 °C for 30 sec, with a final elongation at 72 °C for 5 min.

Nextera XT Index kit V2(illumine, Cat. No: 15052163) was tested as a index primer for Library prep. The PCR product was confirmed by using 1% agarose gel electrophoresis and visualized under a Gel Doc system. The amplified products were purified with the HiAccuBead (AccuGene, Cat. No: ACN01.50). Equal concentrations of purified products were pooled together and removed short fragments (non-target products) with HiAccuBead. Mixed amplicons were pooled and the sequencing was carried out at Asan Institute for Life Science (Seoul, Republic of Korea), with Illumina iSeq100 Sequencing system (Illumina, USA) according to the manufacturer’s instructions. All analyses were performed in EzBioCloud 16S-based MTP, which is a ChunLab’s bioinformatics cloud platform.

To investigate diversity, we calculated alpha diversity (the Chao1, Observed, Shannon, and Simpson Indices) and beta diversity (Principal Coordinate Analysis plot on unweighted UniFrac, weighted UniFrac, Bray-Curtis, and Jaccard Indices). Chao1 values converged toward observed richness in several comparisons because singleton counts were minimal after quality filtering, denoising, and chimera removal. These beta-diversity metrics were selected *a priori* to capture complementary features of community dissimilarity: Bray–Curtis and weighted UniFrac are abundance-weighted, whereas Jaccard and unweighted UniFrac reflect presence/absence structure; UniFrac additionally incorporates phylogenetic relationships. Difference in bacterial taxa level among groups were assessed by linear discriminant analysis (LDA) effect size (LEfSe) and DESeq2 analysis visualized with volcano plot. In the LEfSe analysis, genera with LDA value >2 and *P* <0.05 were regarded as significant. In the DESeq2 analysis, bacterial group with more than 2 log_2_ fold change and *P* <0.05 were regarded as significant.

***Correlation analysis***

To confirm the correlation between gut microbiota and metabolites, we conducted a Spearman correlation analysis. The correlation coefficient was calculated using the cor.test() function in R. **p* < 0.05, ^**^*p* < 0.01 and ^***^*p* < 0.001 were considered statistically significant.

***FMT***

The inoculum for FMT was prepared freshly every transplantation under anaerobic conditions by diluting 1 g of stool sample to 10 mL of sterile PBS and filtered through a 70-µm strainer. Germ-free (GF) C57BL/6 mice were purchased from CLEA Japan Inc. (Tokyo, Japan), bred, and maintained in a sterile isolator until FMT. The mice were housed under controlled temperature (21 ± 1 °C), humidity (45 ± 10%), and lighting (12 h light/dark cycle) and given ad libitum access to sterilized water and standard chow (Teklad 2018S, Envigo, Madison, WI, USA). For FMT, six-week-old male GF mice were randomly divided into 6 groups (*n* = 7~8) and transferred to isolated cages (IsoCage P-Bioexclusion system, TECNIPLAST S.p.A., Buguggiate, Italy): non-emphysema donor 1 (*n* = 7), non-emphysema donor 2 (*n* = 7), non-severe emphysema donor 3 (*n* = 7), non-severe emphysema donor 4 (*n* = 7), severe emphysema donor 5 (*n* = 8), and severe emphysema donor 6 (*n* = 8). No antibiotic pretreatment was used because all recipient mice were germ-free before colonization. For three weeks, each group mice received 200 µL of oral FMT donor-matched inoculations once a week, respectively. Three weeks after, all the FMT mice and their age-matched SPF mice were subjected to smoking exposure. SPF C57BL/6 mice were obtained from two sources, depending on the experimental batch (commercially purchased or house-bred within the facility). All mice were maintained under identical SPF conditions, and experiments were conducted using the same protocol to minimize batch-related variability. All animal experiments were performed in accordance with protocols approved by the Institutional Animal Care and Use Committee of the Asan Medical Center (Permit no. 2022-13-126) and Handong Global University (Permit number: IACUC20221026-14).

***Collection and preparation of mouse samples***

At the end of the experimental period, all mice were anesthetized by inhaled isoflurane and euthanized, and blood samples were collected by cardiac puncture. To collect bronchoalveolar lavage fluid (BALF), the trachea was catheterized and injected with 1.5 mL PBS. To separate supernatants and cellular components, BALF was centrifuged at 2,200 rpm for 5 min at 4°C. The cell pellet was resuspended in PBS, placed on a slide, and stained with Diff-Quick (Sysmex, Kobe, Japan). After BALF collection, lungs were harvested for histology. After ligating the right main bronchus, the left lung lobe was inflated with 0.5% low melting point agarose at a constant pressure of 15 cmH_2_O and fixed in 10% formalin. The remaining right lobe was collected, snap frozen, and stored at -80°C for further analysis. The right postcaval lobe was utilized for quantitative real-time PCR analysis.

***Histopathological and mean linear intercept assessment***

After fixation, each lung lobe was separated, embedded in paraffin, and cut into 5 µm sections. These sections were stained with hematoxylin and eosin to allow measurement of the mean linear intercept (MLI) and thus determine the severity of emphysematous changes. To assess MLI, the mean inter-alveolar septal wall distance was calculated by the number of interruptions in 1 mm lines of the alveolar wall. Four lines were drawn in each field, and at least five random fields per mouse were examined.

***Measurement of cytokine levels***

The levels of tumor necrosis factor (TNF)-α, interferon (IFN)-γ, interleukin (IL)-1, IL-4, IL-6, and IL-12p70 in serum and BALF samples were measured using a commercially available enzyme-linked immunosorbent assay kit (R&D Systems, Minneapolis, MN, USA) according to the manufacturer’s protocols.

***Quantitative real-time PCR***

Total RNA was extracted from lung tissue using TRIzol reagent (Thermo Fisher Scientific, Waltham, MA, USA). Total RNA (1 μg) was used to synthesize cDNA using the primeScript RT Master Mix kit (Takara Bio Inc., Kyoto, Japan). Transcripts were quantified by real-time PCR using sequence-specific primers for TNF-α, IL-6, IFN-γ, and IL-1β. Amplification reactions were performed with Advanced Universal SYBR Green Supermix kit and the CFX Connect Real-Time PCR system (Bio-Rad Laboratories, Hercules, CA, USA). Target gene expression levels were normalized to 18S RNA as an endogenous control gene. The 2^-ΔΔCT^ method was used to calculate the relative changes.

***Bulk RNA sequencing***

A cDNA library was constructed using SureSelectXT RNA Direct Library Preparation kit (Agilent Technologies). Paired end sequencing was performed in the Illumina platform. Generated FASTQ files were further aligned according to GRCh38.v45 primary assembly genome files downloaded from GENCODE using STAR aligner via Nextflow rnaseq (23.10.0). The read count of each gene was calculated using RSEM. Further analysis was performed using the DESeq2 package version 1.42.1 in R. Differentially expressed genes were found using non-normalized count data from RSEM. Gene set enrichment analysis was performed with fgsea version 1.28.0 and msigdbr version 7.5.1 packages in R.

**Supplementary Results**

***Respiratory microbiome analysis of sputum samples***

**Figure S2** shows the sputum microbiome characteristics of the three groups. The alpha-diversity indices of the severe emphysema group were significantly decreased compared to non-emphysema group (**Figure S2a**). The beta-diversity indices also differed significantly among the three groups (**Figure S2b**). Bacterial abundance showed significant differences among the groups, with volcano plots (**Figure S2c**) showing the abundance of *Enterobacteriaceae* and *Moraxellaceae* families in the severe emphysema group. **Figure S3** presents the differential abundance of bacterial groups as determined by LDA. **Figure S4** shows the differential bacterial abundance among the three groups at the genus level. **Table S1** presents specific bacterial groups with significantly differential abundance among the three groups at the phylum, class, order, family, and genus levels.

***Gut microbial abundance in mice following FMT***

The differential abundance of 50 bacterial groups between non-emphysema and non-severe emphysema groups (**Figure S6a**) and 58 bacterial groups between non-emphysema and severe emphysema groups (**Figure S6b**) in the feces were detected using the LEfSe method. **Figure S7** presents taxonomy plots related to the differential bacterial abundance among the five groups at the genus level.

**Table S1**. Differential abundance analysis at genus level in sputum

| **Phylum** | **Class** | **Order** | **Family** | **Genus** |
| --- | --- | --- | --- | --- |
| Firmicutes | Clostridia | Peptostreptococcales-Tissierellales | *Anaerovoracaceae* | *Mogibacterium* |
| Bacteroidota | Bacteroidia | Bacteroidales | *Prevotellaceae* | *Alloprevotella* |
|  |  |  |  | *Prevotella* |
| Actinobacteriota | Actinobacteria | Actinomycetales | *Actinomycetaceae* | *Actinomyces* |
|  | Coriobacteriia | Coriobacteriales | *Atopobiaceae* | *Atopobium* |
| Campilobacterota | Campylobacteria | Campylobacterales | *Campylobacteraceae* | *Campylobacter* |
| Patescibacteria | Saccharimonadia | Saccharimonadales | *Saccharimonadaceae* | *TM7x* |

Bacterial groups at genus level that were significantly abundant in non-emphysema, non-severe emphysema, and severe emphysema groups.

**Supplemental Figure Legends**

**Figure S1.** Flow chart of participant enrollment

Abbreviations: *CT*, computed tomography; *FEV_1_*, forced expiratory volume in 1 second

**Figure S2.** Sputum microbiota characteristics of the three groups

**(a)** Boxplots of alpha-diversity indices (Chao 1, Observed, Shannon, and Simpson Indices). Wilcoxon rank sum test was used. **P* < 0.05, ***P* < 0.01.**(b)** Beta-diversity indices (Principal Coordinates Analysis of unweighted UniFrac, weighted UniFrac, Bray–Curtis, and Jaccard Indices). *P*-value was calculated by PERMANOVA, permutations = 999. **(c)** DESeq2 analysis showing differential bacterial abundance. Bacteria of adjusted *P* <0.05, Log_2_ fold change >2 were presented.

**Figure S3.** Differential bacterial abundance in sputum microbiota

LDA between **(a)** the non-emphysema and non-severe emphysema groups and **(b)** the non-emphysema and severe emphysema groups. Bacterial groups with LDA score >2 and *P* <0.05 are presented. Wilcoxon rank sum test was used.

Abbreviation: *LDA*, linear discriminant analysis

**Figure S4.** Taxonomy bar plot for sputum microbiota

Bacterial groups are presented in order of abundance in non-emphysema, non-severe emphysema, and severe emphysema groups. Wilcoxon rank sum test was used.

**Figure S5.** Beta-diversity analysis of the representative donors from each group

Asterisks in the Principal Coordinates Analysis of unweighted UniFrac indicate the donors in each group. *P*-value was calculated by PERMANOVA, permutations =999.

**Figure S6.** Differential bacterial abundance in fecal samples from mice following FMT

The LDA effect size method for microbiome analysis revealed differential bacterial abundance between **(a)** the non-emphysema and non-severe emphysema groups and **(b)** the non-emphysema and severe emphysema groups. Bacterial groups with LDA score >2 and *P* <0.05 are presented. Wilcoxon rank sum test was used.

Abbreviations: *FMT*, fecal microbiota transplantation; *LDA*, linear discriminant analysis

**Figure S7.** Taxonomy bar plots of differential bacterial abundance in fecal samples from mice following FMT

Bacterial groups, with abundance in the order of severe emphysema, non-severe emphysema, and non-emphysema groups in the green box, and non-emphysema, non-severe emphysema, and severe emphysema in the orange and blue boxes, are presented. Bacterial groups in the blue box were most abundant in the control group. No other statistical analysis was conducted with a set of microorganisms with similar patterns. Some taxa shown at consecutive taxonomic ranks display identical or near-identical abundance patterns because a single descendant lineage dominates the parent lineage.

Abbreviation: *FMT*, fecal microbiota transplantation

**Figure S8.** Correlation between gut microbiota composition and fecal SCFA levels in smoking-exposed germ-free mice following FMT

(a) Heatmap illustrating Spearman correlation coefficients between the relative abundance of gut microbiota (at the family and genus levels) and fecal SCFA concentrations across FMT recipient mice groups. Correlation patterns varied by group: In the non-emphysema-FMT group, *Bacteroides* genus showed a strong positive correlation with fecal acetate levels. In contrast, the non-severe emphysema-FMT group exhibited a positive correlation between *Bifidobacterium* genus and propionate levels. In the severe emphysema-FMT group, members of the *Ruminococcaceae* family were positively correlated with SCFA levels, particularly with butyrate.

(b) Taxon-specific correlation analysis at the species level for microbial genera associated with SCFAs. Among these, *Bacteroides uniformis* demonstrated a strong and specific positive association with fecal acetate concentrations in mice receiving FMT from non-emphysema donors, suggesting a potential mechanistic link between these species and the elevated acetate production observed in this group.

Abbreviations: *FMT*, fecal microbiota transplantation; *SCFA*, short-chain fatty acid.
